# Hermeneutics of the wheel of time: The environmental dance of aged micro- and nanoplastics and their biological resonance

**DOI:** 10.1016/j.clinsp.2025.100767

**Published:** 2025-09-11

**Authors:** Ilker Sengul, Demet Sengul

**Affiliations:** a Thyroidology Unit, Giresun University Faculty of Medicine, Giresun, Turkey; b Division of Endocrine Surgery, Giresun University Faculty of Medicine, Giresun, Turkey; c Department of General Surgery, Giresun University Faculty of Medicine, Giresun, Turkey; d Department of Pathology, Giresun University Faculty of Medicine, Giresun, Turkey

*Dear Editor*,

The effects of micro- and nanoplastics,^1^, ^2^, ^3^, ^4^ *per se*, on tissues and the environment remain crucial for health care providers and environmental scientists. These are born from the larger works of men, which break apart into dust and smaller bits with time and the harsh touch of sun, wind, and water. We have read the article “The wheel of time: The environmental dance of aged micro- and nanoplastics and their biological resonance” with great interest.^5^ These small shards, the writing calls them 'micro-' and 'nano-plastics', journey afar, settling in the great oceans and the very soil beneath our feet. Yet, it is not merely their presence that causes unease, but how they change with the passage of time ‒ a process the writing names 'aging'. The sun alters their faces, the wind scratches them, and the heat shapes them anew. And lo, these aged shards are different! Their surfaces change, making them cling to other things or give forth the hidden substances within them. This alteration is not without consequence for the living world. The authors recount how these shards, especially when aged, bring harm upon the fish of the water, the plants of the field, and even the tiny life within cells. The authors speak of illness in the gut and the body, and how these smallest shards can cross barriers within creatures. However, there are some knowledge gaps regarding the natural aging behaviors of Micro- and Nanoplastics (MNPs), involving the aging mechanisms of different types of plastic, the influence of various environmental factors, and the specific interaction between their thermal and photoaging processes. *Ad hoc*, the release of associated chemicals and additives from MNPs during aging and their specific toxic effects remain areas with limited research. The effects of aging on the biological impacts of MNPs are still relatively unexplored. The effects of aging on the toxicity of MNPs are complex and not fully understood, with even opposite conclusions reported in some studies regarding cytotoxicity and pathology. Truly, this writing shines a light upon a dark concern of our age, which exhibits that the journey of these small shards is shaped by time and the elements, and that this shaping changes how they interact with life and the earth itself.^5^ Yet, the study admits that much is still hidden from our sight; the whole tale of this aging and its profound effects is not yet known. More wisdom is needed to understand this matter fully, so that we might better guard our world and the life upon it. This issue merits further investigation. We thank Li et al.^5^ for their valuable study on MNPs*.*

*Ægroto dum anima est, spes est.*

## Ethics approval

Not applicable.

## Consent to participate

Not applicable.

## Consent to publish

Not applicable.

## Data availability

The datasets used and/or analyzed during this study are available from the corresponding author upon reasonable request.

## Abbreviation

MNPs: Micro- and nanoplastics

## Funding

None declared.

## CRediT authorship contribution statement

**Ilker Sengul:** Conceptualization, Formal analysis, Investigation, Methodology, Project administration, Resources, Software, Validation, Visualization, Writing – original draft, Writing – review & editing. **Demet Sengul:** Conceptualization, Formal analysis, Investigation, Methodology, Project administration, Resources, Software, Supervision, Validation, Visualization, Writing – original draft, Writing – review & editing.

## Declaration of competing interest

The authors declare no conflicts of interest.
